# Highly Efficient Methylene Blue Dye Removal by Nickel Molybdate Nanosorbent

**DOI:** 10.3390/molecules26051378

**Published:** 2021-03-04

**Authors:** Souad Rakass, Hicham Oudghiri Hassani, Ahmed Mohmoud, Fethi Kooli, Mostafa Abboudi, Eman Assirey, Fahd Al Wadaani

**Affiliations:** 1 Laboratory of Applied Organic Chemistry (LCOA), Chemistry Department, Faculty of Sciences and Techniques, Sidi Mohamed Ben Abdellah University, Imouzzer Road, P.O. Box 2202, 30000 Fez, Morocco; 2 Engineering Laboratory of Organometallic, Molecular Materials and Environment (LIMOME), Faculty of Sciences, Chemistry Department, Sidi Mohamed Ben Abdellah University, P.O. Box 1796 (Atlas), 30000 Fez, Morocco; oudghiri_hassani_hicham@yahoo.com; 3 Petroleum Technology, Operated Offshore Oil Field Development, Qatar Petroleum, P.O. Box 3212, Doha, Qatar; caadil77@yahoo.co.uk; 4 Chemistry Department, College of Science, Taibah University, Al-Madinah Al-Munawwarah 30002, Saudi Arabia; abboudi14@hotmail.com (M.A.); eman_assirey@hotmail.com (E.A.); fwadaani@taibahu.edu.sa (F.A.W.); 5 Department of Chemistry, Faculty of Science, Islamic University of Madinah, Al-Madinah Al-Munawwarah 42351, Saudi Arabia; fethi_kooli@yahoo.com

**Keywords:** nanosorbent, regeneration, α-NiMoO_4_, methylene blue, removal

## Abstract

Removing methylene blue (MB) dye from aqueous solutions was examined by the use of nickel molybdate (α-NiMoO_4_) as an adsorbent produced by an uncomplicated, rapid, and cost-effective method. Different results were produced by varying different parameters such as the pH, the adsorbent dose, the temperature, the contact time, and the initial dye concentration. Adsorbent dose and pH had a major removal effect on MB. Interestingly, a lower amount of adsorbent dose caused greater MB removal. The amount of removal gained was efficient and reached a 99% level with an initial methylene blue solution concentration of ≤160 ppm at pH 11. The kinetic studies indicated that the pseudo-second-order kinetic model relates very well with that of the obtained experimental results. The thermodynamic studies showed that removing the MB dye was favorable, spontaneous, and endothermic. Impressively, the highest quantity of removal amount of MB dye was 16,863 mg/g, as shown by the Langmuir model. The thermal regeneration tests revealed that the efficiency of removing MB (11,608 mg/g) was retained following three continuous rounds of recycled adsorbents. Adsorption of MB onto α-NiMoO_4_ nanoparticles and its regeneration were confirmed by Fourier transform infrared spectroscopy (FTIR) analysis and scanning electron microscopy (SEM) analysis. The results indicated that α-NiMoO_4_ nanosorbent is an outstanding and strong candidate that can be used for removing the maximum capacity of MB dye in wastewater.

## 1. Introduction

Dyes have recently been extensively utilized in several industrial and manufacturing applications, e.g., printing, textile, paper, carpet, and cosmetics. Dyes are considered toxic, hazardous pollutants and require removal before their discharge into the environment [1,2,3,4,5,6,7].

Numerous methods have been developed for dye removal from wastewater and industrial waste matter, including adsorption, coagulation, photodegradation, flocculation, membrane separation, ion exchange, biological treatment, chemical oxidation, and extraction [8,9,10,11,12,13,14,15].

Adsorption application is extensively employed due to its ease of process and guarantee of superb minimal cost from among those above-mentioned methods [1,16,17,18,19,20,21]. Several natural adsorbents have been successful in the elimination of color from aqueous waste matter [22,23,24,25]. A universally used example is activated carbon owing to the presence of its large surface area [26,27]. There are still some difficulties that limit its use, such as its high cost of production, low-quality mechanical properties, regeneration issues, and phase separation strain [28]. The challenge faced by the researchers is to develop novel adsorbents with boundless adsorption capabilities that are capable of being regenerated for the recovery of reusable compounds.

In recent years, researchers have shown a great interest in binary metal oxides due to their potential performances for different materials [29]. In recent years, scientists have paid extensively studied the group of metal molybdates which has given the most favorable examples of mixed metal oxides [30,31,32,33]. Nickel molybdate (NiMoO_4_) has various applications in catalysis such as hydrodesulfurization and hydrodenitrogenation reactions [34,35], oxidative dehydrogenation of light alkanes [36,37,38,39,40], partial oxidation of hydrocarbons [41], and microwave applications [42]. It is also used in humidity sensors [43], supercapacitors [44,45], optical fibers, and military devices [46]. Nickel molybdate has attractive structures and electrochemical and magnetic properties [47,48], and it can be found in two crystalline forms, α-NiMoO_4_ and β-NiMoO_4_.

Various methods of NiMoO_4_ synthesis have been presented in the literature, including sonochemical [49,50], hydrothermal [46,51,52], precipitation [53,54], sol–gel [53], mechanochemical synthesis [55], solid state at high temperature [56,57], and microwave-assisted methods [58].

Recently, molybdate compounds have attracted great interest for their utilization in environmental applications such as the photocatalytic oxidation of dyes [59,60,61,62], the oxidation of methylene blue (MB) dye [63], and the sorption of water-soluble dyes [30,61].

In particular, α-NiMoO_4_ synthesized by the microwave-assisted method has shown good photocatalytic activity for methylene blue photodegradation [58]. In addition, NiMoO_4_ nanostructures synthesized by the coprecipitation method were efficiently used as a catalyst for methyl orange photooxidation under UV irradiation [64]. Furthermore, hydrothermally synthesized β-NiMoO_4_ was recently used as a sono-photocatalyst for the degradation of methylene blue (MB) under diffused sunlight [65]. However, α-NiMoO_4_ has not yet been explored for the removal of dyes by adsorption.

In the present work, nickel molybdate nanoparticles, synthesized using a facile and easy method without the use of any solvents, were evaluated for removing methylene blue dye (MB) as adsorbents. MB dye was utilized as an ideal dye owing to its extensive manufacturing uses as a food coloring agent and for cotton, wool, silk, and leather, to name a few examples [60]. The influence of diverse parameters, namely solution pH, initial concentration, adsorbent dose, and contact time, on the removal of methylene blue by synthesized α-NiMoO_4_ nanosorbents was examined. The kinetics and adsorption isotherms were evaluated. In addition, after the nanosorbent had been regenerated by calcinating at high temperature, the removal efficiency was likewise investigated.

## 2. Results and Discussion

### 2.1. Removal of MB

#### 2.1.1. pH Point of Zero Charge (pH_pzc_)

**Figure 1:**
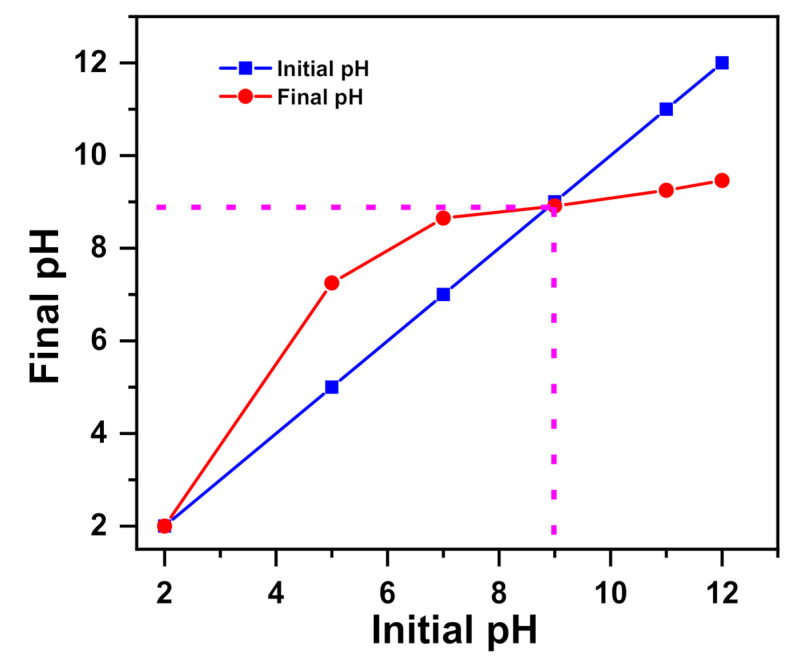
pH_pzc_ for nickel molybdate.

The pH point of zero charge (pH_pzc_) can provide information regarding the surface charge of a material. The results found for this parameter are given in Figure 1. In fact, the pH_pzc_ of nickel molybdate was determined from graphs where the initial pH is equal to the final pH (intersection of curves). As shown in Figure 1, the nickel molybdate presented a surface charged negatively (pH_pzc_ = 8.96). The surface of nickel molybdate became negatively charged for a pH of solutions >8.96 and acquired a positive charge when the pH was lower than 8.96. According to the literature, the cation uptake is favorable at a pH > pH_pzc_, whereas the uptake of anions is encouraged at a pH < pH_pzc_ of sorbent [66]. The obtained pH point of zero charge value is close to those obtained for some metal oxides such as CuO and NiO, which are in the range of 9–10 [67].

#### 2.1.2. Effect of pH

**Figure 2:**
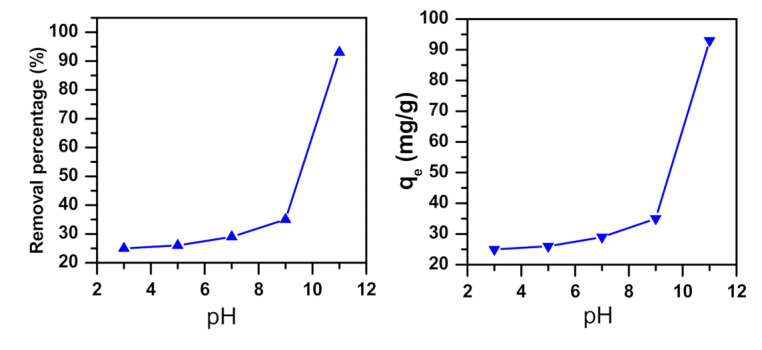
Effect of pH on dye removal performance of α-NiMoO_4_ in a 100 ppm methylene blue solution (m_ads_ = 0.1 g, T = 20 °C, t = 30 min).

pH is essential in terms of controlling the removal of dyes. It has no effect on altering the separation of the adsorbent site; nevertheless, it modifies the structure and the chemistry of the dye [68]. Moreover, the charge and surface potential of the oxide are primarily pH-dependent. It is possible, in some cases, to adjust the pH conditions of the slurry in such a way that all the particles exhibit the same charge polarity [68]. Hence, the effect of pH on the removal of MB by nickel molybdate (α-NiMoO_4_) nanosorbent was assessed by varying pH values between 3 and 11 at a controlled temperature of 20 °C with the initial concentration of 100 ppm. Figure 2 demonstrates that methylene blue removal depended on the effect of pH. By increasing the pH from 3 to 7, the removal percentage did not substantially change and remained at about 29%. The further increase of pH to 9 slightly increased the removal percentage to 35%. The increase of pH to 11 led to the highly efficient removal of MB, as the removal percentage reached 93%. Moreover, there was an increase in the quantity of dye eliminated for every unit mass of the adsorbent at its equilibrium (qe) from 25 to 93 mg/g.

Strong electrostatic interactions took place between the charges of the MB dyes and those of α-NiMoO_4_ adsorbent, as demonstrated by the increase in removal percentage obtained with the increase of the pH values. Furthermore, in the solution at pH 11, the hydroxyl group (OH^−^) favors the direction of the positively charged MB as the pKa equals 3.8 [69]. Nevertheless, the lower removal performance at acidic values may well be related to the extra proton ions within the solution which are in concurrency with those of the basic dye cations on the removal sites of α-NiMoO_4_. Comparable outcomes were reported by Kooli et al. in the examination of waste bricks utilized as favorable agents for the removal of basic blue 41 from liquid solutions [70].

On the other hand, these results can be explained by the point of zero charge value measured for nickel molybdate (pH_pzc_ = 8.96). The literature reports that at lower pH (pH < pH_pzc_), the surface charge may become positive, thus allowing H^+^ ions to compete effectively with dye cations and causing a decrease in the amount of dye adsorbed [71]. At higher pH (pH > pH_pzc_), the nickel molybdate may become negatively charged, which enhances the positively charged cationic dye through electrostatic forces of attraction.

Thus, pH 11 was found to be the best value for the removal of MB when employing α-NiMoO_4_ nanosorbent.

#### 2.1.3. Effect of Adsorbent Dose

**Figure 3:**
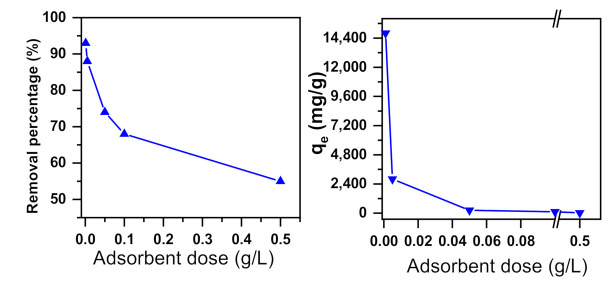
Effect of adsorbent dose on the dye removal performance of α-NiMoO_4_ in a 160 ppm methylene blue solution (t = 30 min, T = 20 °C).

One crucial parameter in adsorption processes is the adsorbent dose [72]. The MB dye removal using α-NiMoO_4_ was explored with varying adsorbent doses of 0.001 to 0.5 g/L with an initial dye concentration of 160 ppm. As can be seen clearly in Figure 3, the percentage (%) and the amount (mg/g) of MB removed were reduced as the nanosorbent dose increased from 0.001 to 0.5 g/L. The decrease in the removal effectiveness might be due to the performance of particle interaction (e.g., aggregation) as a result of the high dosage of the adsorbent. Such aggregation could increase diffusional path duration. Furthermore, the adsorption sites remain unsaturated throughout the course of sorption under these conditions. In fact, all of these factors can lead to a decrease in available particle size [73,74].

#### 2.1.4. Effects of Initial Concentration and Contact Time

**Figure 4:**
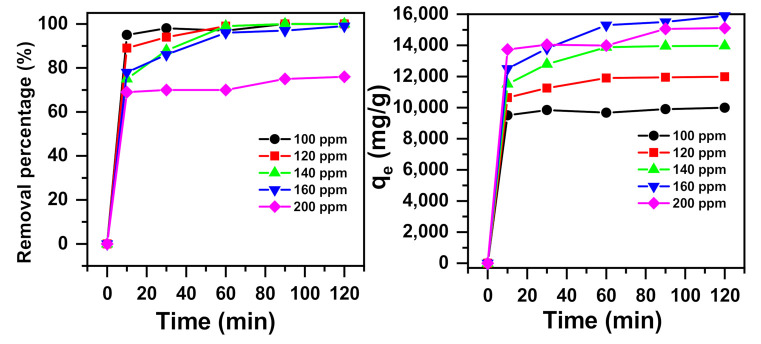
Impact of the initial dye concentration and contact time on the methylene blue (MB) dye removal performance of α-NiMoO_4_ (m_adsorbent_ = 0.001 g, T = 20°C, pH = 11).

Figure 4 presents the effects of initial MB dye concentration and contact time on dye removal. The removal of MB was enhanced with the increase in contact time, reaching the highest value of 98% at around 30 min for initial methylene blue concentrations of 100 ppm. For MB concentrations of 120, 140, and 160 ppm, a maximum value of 99% removal was reached at around 60 min. However, the removal percentage of MB decreased from 99% to 76% as the Ci value was increased from 160 to 200 ppm. The removal amount remarkably increased from 9993 mg/g to 15,900 mg/g when the initial dye concentration increased from 100 to 160 ppm and remained stable when the concentration was increased to 200 ppm. This showed that the concentration gradient is an essential factor that drives the overcoming of the mass transfer resistances within the solid and liquid phases. The ratio of the solution connected with the α-NiMoO_4_ surface was higher at lower MB concentrations, which triggered a rise in removal efficiency. In contrast, at higher MB dye concentrations, the decrease in the adsorption percentage was affected by the saturation of active sites on the α-NiMoO_4_ surface [75].

#### 2.1.5. Temperature Effect

**Figure 5:**
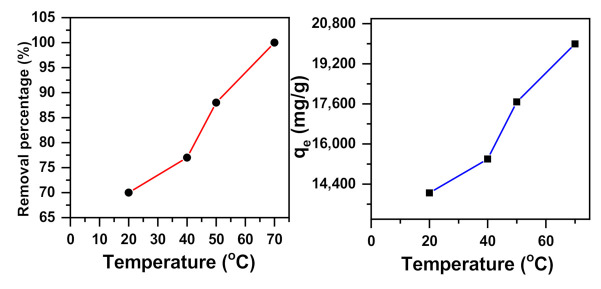
The effect of temperature on the dye removal capacity of NiMoO_4_ in a 200 ppm methylene blue solution (t = 30 min, pH = 11).

The temperature is an essential factor that greatly affects the removal of dyes [76]. The procedure for removing the methylene blue dye was examined from 20 to 70 °C, as shown in Figure 5. The outcome of temperature experiments shows that the removal percentage increased from 70% to 100% and the removal capacity increased from 14,047 to 19,990 mg/g at an initial MB dye concentration of 200 ppm. The efficiency progression of MB removal with a rise in temperature was due to the intensity of attractive forces between removal sites and the MB, which shows an endothermic process [70]. In addition, increasing the temperature improves the removal motion of the adsorbent sites and the dye molecule motion [76].

**Figure 6:**
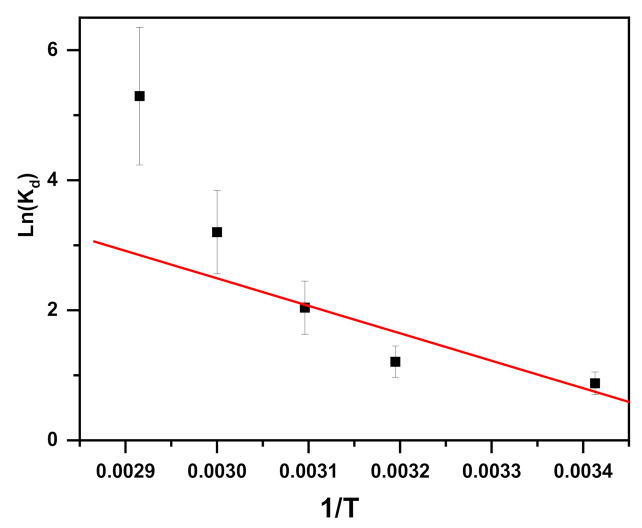
Van’t Hoff plot presenting the impact of temperature on methylene blue dye removal utilizing α-NiMoO_4_.

Thermodynamic factors are also important factors in adsorption processes [77,78]. The probability and the mechanism of adsorption can be predicted by the thermodynamic factors [77]. The following equations are used to determine the thermodynamic parameters:

(1) $$\Delta G^{o}=-{\mathrm{RTLnK}}_{d}$$

(2) $$K_{d}=\frac{C_{a}}{C_{e}}$$

(3) $${\mathrm{LnK}}_{d}=\frac{\Delta S^{o}}{R}-\frac{H^{o}}{\mathrm{RT}}$$

where R is the gas constant (J mol^−1^ K^−1^), ΔG° is the free energy, K_d_ is the distribution constant, T is absolute temperature (K), C_a_ is the quantity of dye adsorbed by the adsorbent at equilibrium (mol/L), C_e_ is the equilibrium concentration, ΔH° is the standard enthalpy, and ΔS° is the standard entropy. ∆S° and ∆H° values were obtained from the intercept and slope of the ln K_d_ versus 1/T plot (Figure 6). ∆G° values were obtained from Equation (1) and are shown in Table 1. The adsorption is favorable and spontaneous, and this is revealed by the negative value obtained for ∆G°. In fact, Gibbs free energy change (Δ*G*°) values can discern whether a process is spontaneous or not, and negative values of Δ*G*° imply a spontaneous process. The enthalpy change (Δ*H*°) provides information about the exothermic or endothermic nature of the process and differentiates between physical and chemical adsorption processes. Therefore, the positive value of ∆H° (35.12 kJ mol^−1^) shows that methylene blue removal followed an exothermic process. In addition, the (∆H°) value was found to be less than 40 kJ/ mol, which indicates that the adsorption of MB by nickel molybdate is physisorption [79]. The present results are similar to the results reported by Xia [80] for adsorption of congo red from aqueous solution by CTAB–hectorite and ODA–hectorite composites. The enhanced anarchy and uncertainty in the solid solution interface of methylene blue and α-NiMoO_4_ are shown by the positive values of ∆S°. The adsorbate molecules move the adsorbed water molecules; consequently, translational energy is gained rather than lost, which indicates that this approach takes place randomly [81].

### 2.2. Kinetic Study

Kinetic study of removal of methylene blue dye has been conducted as it provides an indication regarding the adsorption system [82].

The data found from the kinetics of removing MB dye using α-NiMoO_4_ nanosorbent were examined by pseudo-first-order, pseudo-second-order, and intraparticle diffusion models. Equations of the studied models are shown in Table 2.

**Figure 7:**
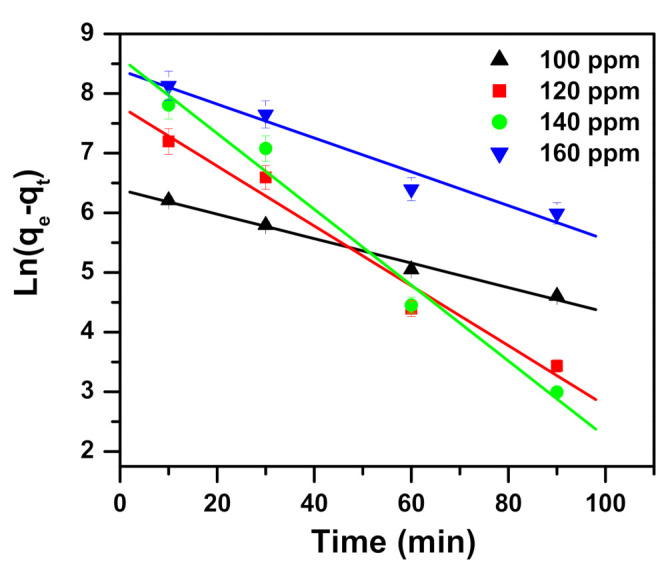
Pseudo-first-order model plot showing the impact of contact time and initial dye concentration on methylene blue removal utilizing α-NiMoO_4_.

**Figure 8:**
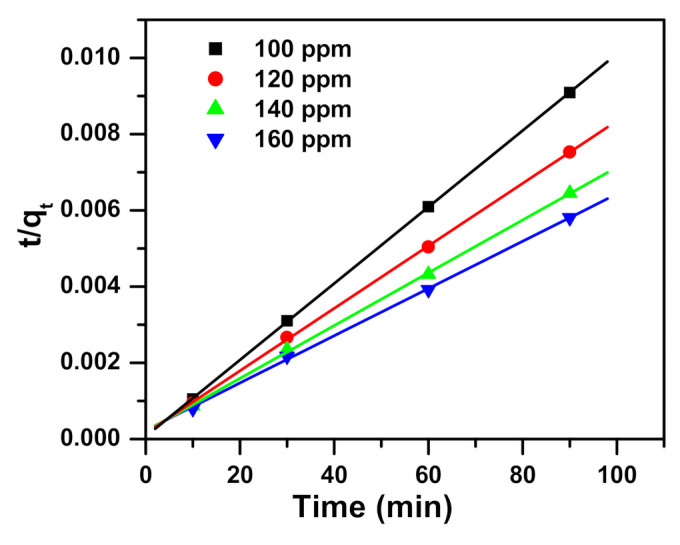
Pseudo-second-order model plot showing the impact of contact time and initial dye concentration on methylene blue dye removal utilizing α-NiMoO_4_.

**Figure 9:**
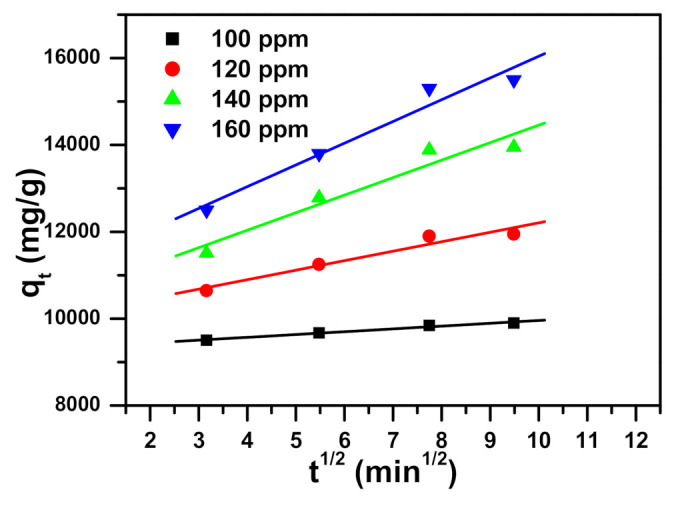
Intraparticle diffusion model plot showing the impact of contact time and initial dye concentration on methylene blue dye removal utilizing α-NiMoO_4_.

Three model parameters, namely pseudo-first-order, pseudo-second-order, and intraparticle diffusion, are presented in Table 3 and displayed in Figure 7, Figure 8, Figure 9 respectively. Regression correlation coefficients (R^2^) of the three models vary. Intraparticle diffusion is 0.934 to 0.981, pseudo-first-order ranges from 0.952 to 0.988, and pseudo-second-order is 0.999 to 1.000, varying with their concentrations used. The R^2^ for pseudo-second-order is equal to or near 1, and hence this model fits very well.

### 2.3. Adsorption Isotherms

When planning adsorption methods, adsorption isotherms, which are known to be essential, are taken into consideration due to their perfect explanation [84]. Four adsorption models have been examined, namely Dubinin–Radushkevich, Temkin, Freundlich, and Langmuir models. Equations of the four examined models are presented in Table 4.

**Figure 10:**
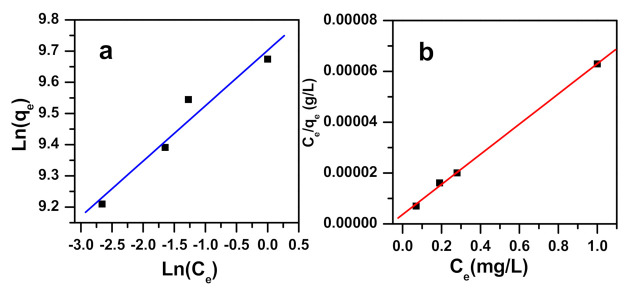
Freundlich (**a**) and Langmuir (**b**) isotherm model plots presenting the results of initial dye concentration and the removal of methylene blue dye utilizing α-NiMoO_4_.

The models employed to match the investigational data were Freundlich, Langmuir, Temkin, and D–R isotherm. Standards of regression correlation coefficients (R^2^) and the model parameters are displayed in Figure 10 and contained in Table 5. Langmuir equation demonstrated the highest value of R^2^ (0.999), and D–R model revealed the lowest value of R^2^ (0.782), while intermediary values were attained for Temkin and Freundlich (0.960 and 0.948, respectively). The Langmuir model fits the experimental results well; the methylene blue removal occurred on a homogeneous surface, establishing a monolayer on the α-NiMoO_4_ adsorbent, with a high adsorption capacity of 16,863 mg/g. Methylene blue dye removal by α-NiMoO_4_ is favorable and is revealed by the RL separation factor ranging from 0.0004 to 0.0006.

Table 6 presents previous reports of the maximum amount of methylene blue dye removed. When compared with many nanosorbents, Nickel-based nanosorbents NiO (Q_max_ = 10,585 mg/g) and α-NiMoO_4_ (Q_max_ = 16,863.00 mg/g) show a considerably higher rate of adsorption for MB. The adsorption capacity of Fe_2_(MoO_4_)_3_ (Q_max_ = 6173.00 mg/g) is lower than that obtained by α-NiMoO_4_, which can be related to the difference in their specific surface area (8.03 versus 29.86 m^2^/g). Thus, α-NiMoO_4_ has the advantage of being able to be synthesized at a rather low temperature via a relatively cost-effective, very simple procedure for use in potential novel, more efficient decontamination processes aimed at the removal of methylene pollutants.

### 2.4. Regeneration and Characterization of the α-NiMoO_4_ Nanosorbent

#### 2.4.1. Regeneration Performance

The repeatability and regeneration of nanosorbents are quite important for their practical applications. Regeneration techniques suggested in the literature include chemical extraction, thermal treatment, supercritical regeneration, microwave irradiation, bio-regeneration, etc. [25,88,93,94,95]. The thermal regeneration, which has been applied for molybdenum oxide nanosorbent, was described in our previously published work [88]. In this investigation, the thermal treatment technique was examined for purposes of regeneration testing, as the structure of the α-NiMoO_4_ removal agent was steady. The adsorbed MB was completely oxidized and decomposed during the calcination process.

**Figure 11:**
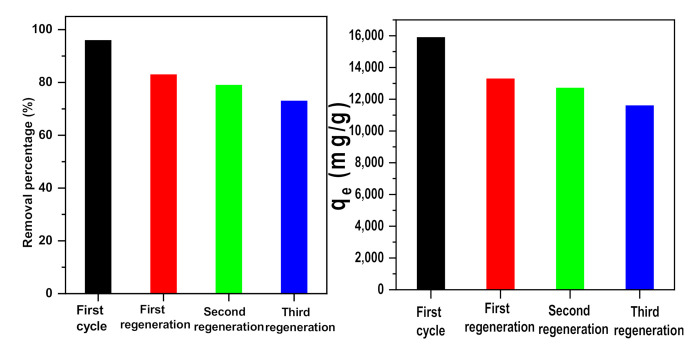
Recycled performance of α-NiMoO_4_ in the removal of MB dye.

The results showed that α-NiMoO_4_ could be re-stimulated through thermal treatment. Figure 11 indicates the reused performance of α-NiMoO_4_ in the removal of MB in three cycles. As a matter of fact, the data show a decrease in dye removal from 99% to 73% with a decrease in removal capacity from 15,900 to 11,608mg/g. The maximum adsorption capacity obtained after four cycles of use (11,608 mg/g) was higher than that obtained by several nanosorbents [30,87,88,89,90,91,92]. The high level of removal efficiency showed that the adsorbent regeneration by way of calcination under atmospheric air at a temperature of 400 °C was extremely efficient and indicative of outstanding recycling capability.

#### 2.4.2. Fourier Transform Infrared Spectroscopy

**Figure 12:**
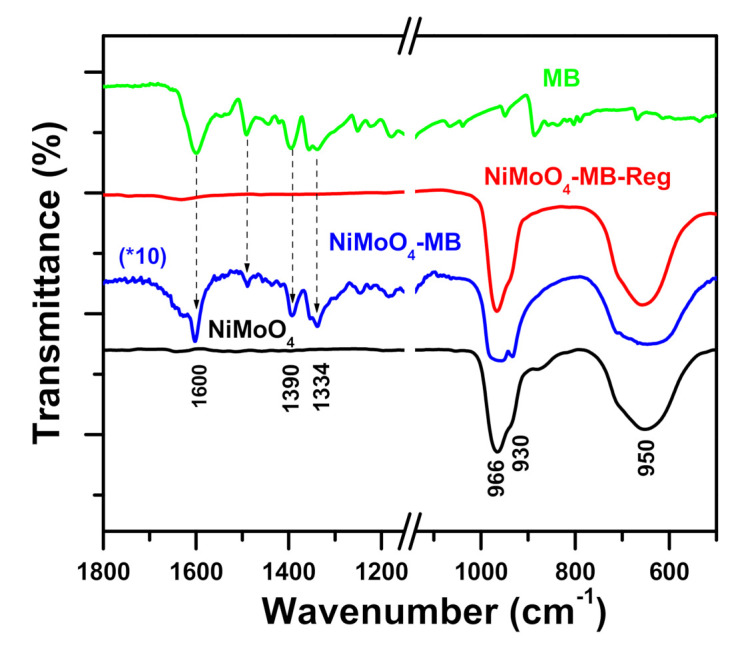
Fourier transform infrared spectra of NiMoO_4_, NiMoO_4_-MB, NiMoO_4_-MB-Reg, and MB.

To completely recognize the method by which α-NiMoO_4_ nanosorbent removes MB dye, the components subjected to MB dye were examined by FT-IR spectroscopy. Figure 12 shows the FTIR spectra for the α-NiMoO_4_ sample before and after the removal of methylene blue dye. As observed, the characteristics of flexing and stretching vibrations of the metal–oxygen bonds at 966 and 930 cm^−1^ and the broad, centered bonds at 650 cm^−1^ correspond to nickel molybdate [96]. The FTIR spectrum of the pure methylene blue displayed bands between 1700 and 1000 cm^−1^ [97]. The FTIR spectrum of NiMoO_4_ after adsorption of methylene blue (NiMoO_4_-MB) displayed further bands located at 1600 cm^−1^, related to the C=C stretching of methylene blue, because of the presence of the methylene blue attached to the active sites of NiMoO_4_ [98]. The FTIR spectrum of the regenerated NiMoO_4_ (NiMoO_4_-MB-Reg) after thermal treatment and the FTIR spectrum of fresh NiMoO_4_ were alike, indicating thorough combustion of the attached methylene blue on the surface, and the resulting spectrum showed the cleanness and performance of the regenerated adsorbent.

### 2.5. Removal Mechanism of MB

**Figure 13:**
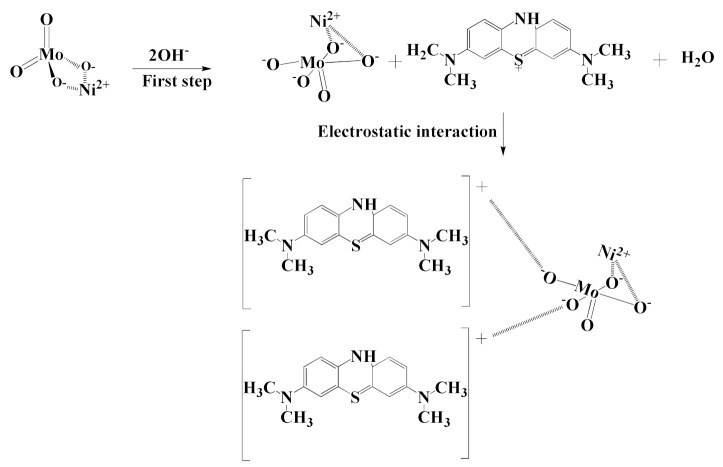
Schematic mechanism of the methylene blue dye removal by α-NiMoO_4_ nanosorbent.

The removal of MB by α-NiMoO_4_ nanoparticles was discovered to be due to the adsorption mechanism. Moreover, FTIR spectroscopy revealed that the removed methylene blue cations were triggered by the adsorption method without any intermediate compounds produced due to the absence of MB decomposition. Additionally, by using α-NiMoO_4_ nanoparticles, the effectiveness of MB dye removal increased with the increase in pH up to pH 11, and this may be credited to its basic media. A reasonable mechanism can be proposed (Figure 13) on the basis of these findings. Furthermore, the positive charge of the MB dye is sustained in the first step at pH 11 since the pKa is equal to 3.8 [69]. Additionally, α-NiMoO_4_ reacts with the hydroxyl groups (OH^−^) in the solution to generate the ion nickel molybdate (NiMoO_5_^2−^) with no intermediate compounds present [99]. Hence, the adsorption is directed by the strong electrostatic interactions between the negatively charged surface of nickel molybdate (NiMoO_5_^2−^) and the positive charge of methylene blue cations [88].

**Figure 14:**
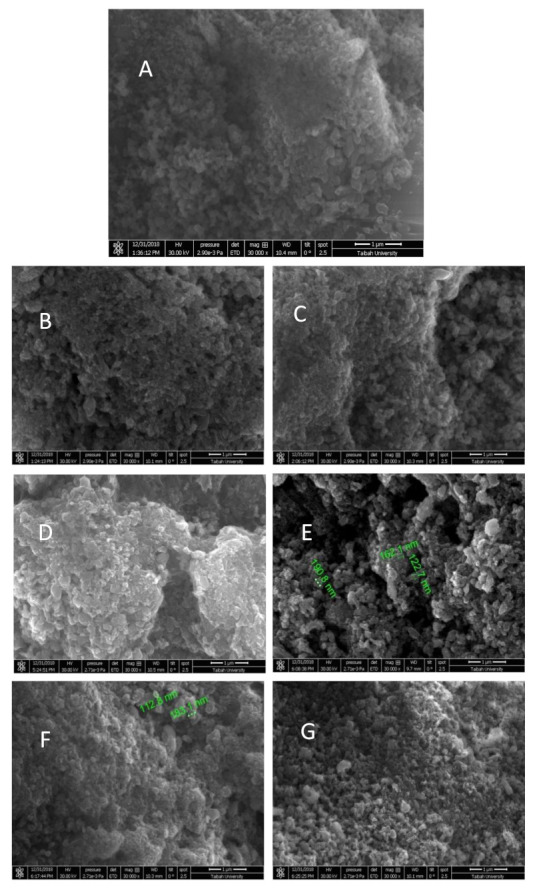
SEM micrographs: (**A**) the starting material, pure nickel molybdate (α-NiMoO_4_); (**B**) the material after the MB dye had been removed; (**C**) the regenerated α-NiMoO_4_; (**D**) the material after the second regeneration and/or removal cycle of the methylene blue dye; (**E**) the morphology of α-NiMoO_4_ after the second regeneration process; (**F**) the material after the third regeneration/removal cycle of methylene blue dye; (**G**) the morphology of α-NiMoO_4_ after the third regeneration.

It is important to adhere to the progression of the α-NiMoO_4_ morphology at different phases of the adsorption study. The SEM micrograph in Figure 14A gives an idea about how the particles form aggregates, showing a good porosity that can allow the improved adsorption of the dye. Nevertheless, the micrographs in Figure 14B,D,F show much less porous powder after the adsorption experiments; the methylene blue molecules filled the pores present in the starting samples. Figure 14C,E,G shows that the morphology of the sample did not change after the regeneration or the first and second reuses. In all cases, the particles were less agglomerated, displaying exceptionally porous powder. The morphology of α-NiMoO_4_ was not significantly altered, even after the second and third reuses (Figure 14E,G).

## 3. Materials and Methods

### 3.1. Nickel Molybdate Nanosorbent Preparation

All synthetic compounds aside from the methylene blue (provided by Panreac, Barcelona, Spain) were purchased from Sigma-Aldrich (St. Louis, MO, USA) and utilized as received with no alterations.

Nickel molybdate (NiMoO_4_) was formed by thermal breakdown of a nickel molybdenum complex obtained from the reaction of oxalic acid dihydrate H_2_C_2_O_4_·2H_2_O, nickel nitrate Ni(NO_3_)_2_·6H_2_O, and ammonium molybdate (NH_4_)_6_Mo_7_O_24_·4H_2_O in its solid form, as defined previously in the literature [32]. Nickel nitrate, oxalic acid dihydrate, and ammonium molybdate were blended jointly together in a molar proportion of 1/10/0.143. The mixture was powdered homogeneously and placed on a hot plate at a temperature of 160 °C for heating. The obtained nickel molybdenum complex was then decomposed under the control with static air at 500 °C for 2 h inside a cylindrical furnace, which was open at the two ends.

### 3.2. Adsorption Experiments

Experimental adsorption batches were set up for the removal of the MB dye [75]. The elimination of MB dye by NiMoO_4_ was undertaken by the constant stirring of a precise quantity of adsorbent into a 100 mL MB dye solution with known concentrations and varied temperatures (such as T = 20, 50, and 70 °C) with various contact times (such as 10, 30, 60, 90, and 120 min). Toward the end of prearranged time intervals, before examination with a UV-Visible spectrometer, 0.22 µm syringe filters (Whatman) were used for filtration. Using 0.01 N NaOH or 0.01 N HCl, the pH of the methylene blue solution was easily adjusted. The removed quantity and percentage (%) of methylene blue dye at its equilibrium q_e_ (mg/g) were calculated by the following equations:

(13) $$\mathrm{Removal}\;\%=\frac{C_{0}-C_{e}}{C_{0}}\times 100$$

(14) $$q_{e}=\frac{\left(C_{0}-C_{e}\right)}{M}\times \;V$$

where M stands for the mass of α-NiMoO_4_ (g) added; V is the quantity of solution used (L); and C_e_ and C_0_ are equilibrium and initial concentrations of MB (ppm), respectively. The results were tested three consecutive times, and the percentage uncertainty was found to be around 3%.

### 3.3. Adsorbent Regeneration Method

A solution of 160 ppm was used for regeneration experiments, and the removal equilibrium period was extended by 1 h. The fresh α-NiMoO_4_ used was filtered and dried under a constant temperature of 100 °C and then calcined at 400 °C for 1 h under atmospheric air. The calcined α-NiMoO_4_ was examined to assess the recycling objectives under conditions similar to those for the freshly used α-NiMoO_4_. Once the first recycling test worked well, the restoration process was replicated for three consecutive cycles under consistent conditions.

### 3.4. pH Point of Zero Charge (pH_pzc_) Measure

The pH point of zero charge (pHpzc) of the material was measured by the electrochemical method reported by Altenor et al. [100]. First, 50 mL of a 0.01 M NaCl solution was placed in a 100 mL beaker. Then, the pH was adjusted to successive initial values between 2 and 12 by using either NaOH or HCl (0.1 M), and 0.001 g of nickel molybdate was added to the solutions. After a contact time of 24 h, the final pH was measured and plotted against the initial pH.

### 3.5. Characterization

**Figure 15:**
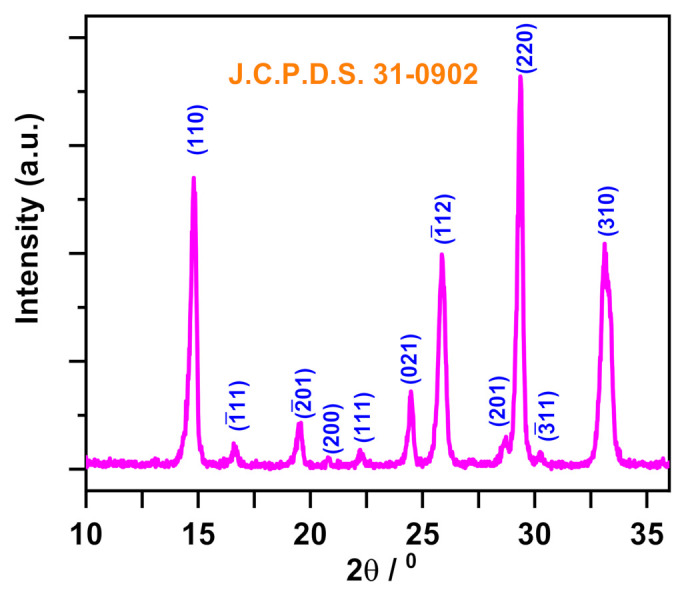
X-ray diffraction pattern of the synthesized NiMoO_4_ nanoparticle powder. The Joint Committee on Powder Diffraction Standards (J.C.P.D.S) index file number is 31-0902.

Analysis of XRD patterns (X-ray diffractometer 6000, Shimadzu, Tokyo, Japan, installed with λ_Cu-Kα_ = 1.5406 Ǻ and Ni filter) was conducted to characterize the phase composition of the synthesized α-NiMoO_4_ nanosorbent, as presented in Figure 15.

The specific surface area characterization was completed by using nitrogen isotherm adsorption in the same way as stated in our previous research work [32]. The recorded specific surface area was 29.86 m^2^/g.

FTIR spectroscopy in the range of 400 to 4000 cm^−1^ (IR Affinity-1S Shimadzu apparatus, Tokyo, Japan) using KBr pellets confirmed the existence of methylene blue dye on α-NiMoO_4_ nanoparticles following the experimental adsorption and regeneration studies.

SEM analysis (Quanta Feg 250, Thermo Fisher Scientific, Hillsboro, OR, USA) was conducted. UV-Visible spectrophotometer (Thermo Scientific Genesys 10S, Madison, WI, USA) was used to determine the concentration at equilibrium.

## 4. Conclusions

α-NiMoO_4_ nanosorbent was synthesized and investigated as a material for the removal of MB dye from aqueous solutions. Removal of MB was extremely reliant on the pH, and this resulted in the achievement of 99% removal efficiency for initial dye concentrations between 100 and 160 ppm at pH 11. The kinetic findings suggested that the removal of methylene blue followed the pseudo-second-order model, and the equilibrium adsorption results were best fitted with the Langmuir isotherm model. The highest removal amount achieved was 16,863 mg/g, as determined by the Langmuir model. Calcination at 400 °C was effectively sufficient to regenerate the adsorbent for further reuse. Even after three cycles of reusing the adsorbent, the MB removal efficiency of NiMoO_4_ was still high. The data proved that α-NiMoO_4_ could be an effective nanosorbent offering outstanding performance in removing MB dye even after being recycled.

## Figures and Tables

**Table 1:** Thermodynamic parameters for the removal of MB dye utilizing α-NiMoO_4_.

Adsorbent	Adsorbate	Temperature (K)	K_d_	∆H° (kJ mol^−1^)	∆S° (kJ mol^−1^ K)		∆G° (kJ mol^−1^)			
α-NiMoO_4_	MB	293	2.401	35.12	0.126	293K	313K	323K	333K	343K
		313	3.348							
		323	7.696							
		333	23.390			−2.134	−3.144	−5.480	−8.727	−14.822
		343	180.818							

**Table 2:** Kinetic model equations.

Model	Equation	Parameters
Pseudo-first-order (PFD) [83]	(4) $\mathrm{Ln}\left(q_{e}-q_{t}\right)={\mathrm{Lnq}}_{e}+K_{1}t$	K_1_: the rate constant of pseudo-first-order adsorption (1/min) q_e_: the removal capacity at equilibrium (mg/g) q_t_: the removal capacity at time t (mg/g)
Pseudo-second-order (PSD) [83]	(5) $\frac{t}{q_{t}}=\frac{1}{K_{2}q_{e}^{2}}+\frac{t}{q_{e}}$	K_2_: the pseudo-second-order rate constant (g·mg^−1^·min^−1^) q_e_: the removal capacity at equilibrium (mg/g) q_t_: the removal capacity at time t (mg/g)
Intraparticle diffusion (IPD) [77]	(6) $q_{t}=K_{I}t^{0.5}+l$	q_t_: the removal capacity (mg/g) at time t t: the contact time (min) I (mg/g) and K_I_ (mg/(g·min^0.5^)): the intraparticle diffusion constants

**Table 3:** Kinetic parameters for removal of methylene blue utilizing α-NiMoO_4_.

C_i_mg/L	Pseudo-First-Order				Pseudo-Second-Order			Intraparticle Diffusion Model		
	q_exp_ (mg/g)	q_e_ (mg/g)	k_1_ (1/min)	R_1_^2^	q_e_ (mg/g)	k_2_ (g/mg min)	R_2_^2^	I (mg/g)	k_i_ (mg/g min^0.5^)	R_3_^2^
100	10,000	584	0.020	0.988	9964	0.00015	1.000	9311	65	0.981
120	11,981	2101	0.048	0.966	12,200	0.00004	1.000	10,027	218	0.946
140	13,970	4770	0.062	0.982	14,436	0.00002	1.000	10,426	403	0.934
160	15,900	4218	0.028	0.952	16,136	0.00002	0.999	11,036	501	0.959

**Table 4:** Adsorption isotherm models for the removal of methylene blue dye utilizing α-NiMoO_4_.

Model	Equation	Parameters
Freundlich [78]	(7) ${\mathrm{Lnq}}_{e}={\mathrm{Lnq}}_{F}+\frac{1}{n}{\mathrm{LnC}}_{e}\;$	C_e_: concentration of MB at equilibrium (ppm) n: the heterogeneity factor (g/L) q_F_: the Freundlich constant (mg^(1−1/n)^ L^1/n^ g^−1^) q_e_: the methylene blue dye quantity adsorbed by α-NiMoO4 at equilibrium (mg/g)
Langmuir [85]	(8) $\frac{C_{e}}{q_{e}}=\frac{1}{q_{m}K_{L}}+\frac{C_{e}}{q_{m}}$	q_e_: the methylene blue dye quantity adsorbed by α-NiMoO_4_ at equilibrium (mg/g) C_e_: concentration of MB at equilibrium (ppm) q_m_: the maximum quantity of methylene blue dye removed by α-NiMoO_4_ (mg/g) K_L_: Langmuir constant of adsorption (L/mg)
	(9) $R_{L}=\frac{1}{1+K_{L}C_{i}}$	C_i_: the initial concentration of methylene blue K_L_: the Langmuir constant R_L_: values indicate that the removal of methylene blue dye could be linear (R_L_ = 1), irreversible (R_L_ = 0), favorable (0 < R_L_< 1), or unfavorable (R_L_ > 1)
Dubinin–Radushkevich (D-R) [84]	(10) ${\mathrm{Lnq}}_{e}={\mathrm{Lnq}}_{m}-{K\varepsilon }^{2}$ (11) $\varepsilon =RTLn\left(1+\frac{1}{C_{e}}\right)$	K: constant for the sorption energy (mol^2^/kJ^2^) ε: the Polanyi potential T: the temperature (K) R: the universal gas constant (8.314 J.mol^−1^ K^−1^) q_m_: the theoretical saturation capacity C_e_: the equilibrium concentration of the methylene blue dye left in the solution (ppm)
Temkin [86]	(12) $q_{e}=B_{T}{\mathrm{LnA}}_{T}+B_{T}{\mathrm{LnC}}_{e}$	b_T_: the Temkin constant related to heat of sorption (J/mol) B_T_ = R_T_/b_T_ R: the gas constant (8.314 J/mol K) A_T_: the Temkin isotherm constant (L/g) T: the absolute temperature (K)

**Table 5:** Isotherm parameters for the removal of MB dye utilizing α-NiMoO_4_.

Langmuir				Freundlich			Temkin			Dubinin–Radushkevich		
q_m_ (mg/g)	K_L_ (L/ mg)	R^2^	Range R_L_	q_F_(mg^(1−1/n)^L^1/n^g^−1^)	1/n	R^2^	A_T_ (L/g)	B_T_	R^2^	q_m_ (mg/g)	R^2^	E (Kj/ mol)
16,863	16	0.999	0.0004–0.0006	16,318	6	0.948	1220	2269	0.960	14,429	0.782	43

**Table 6:** Previous reports of the maximum amount of methylene blue dye removed (q_m_).

Nanosorbent	Q_max_ (mg/g)	Reference
ZnMoO_4_ nanoparticles	217.86	[30]
Fe_2_(MoO_4_)_3_ nanoparticles	6173.00	[87]
α-MoO_3_ nanoparticles	152.00	[88]
Chemically reduced graphene oxide	1519.60	[89]
Magnetic β-cyclodextrin–chitosan nanoparticles	2783.30	[90]
ZnO	7918.02–9197.70	[91]
Fe_2_O_3_	1124.70	[92]
CoO	5501.93	[92]
NiO	10,585.00	[92]
Nickel molybdate (α-NiMoO_4_)	16,863.00	This work

## Data Availability

The data that support the findings of this study are available from the corresponding author upon reasonable request.
